# Sexual Satisfaction After Gender Affirmation Surgery in Transgender Individuals

**DOI:** 10.7759/cureus.27365

**Published:** 2022-07-27

**Authors:** Renard R Jerome, Maneesha K Randhawa, John Kowalczyk, Alexander Sinclair, Ishita Monga

**Affiliations:** 1 Internal Medicine, St. George's University School of Medicine, St. George, GRD; 2 Psychiatry, St. George's University School of Medicine, St. George, GRD; 3 Surgery, Southern California Hospital, Los Angeles, USA; 4 Biological Sciences, University of California Los Angeles, Los Angeles, USA

**Keywords:** male-to-female, gender-dysphoria, gender affirmation surgery, sexual-satisfaction, transgender

## Abstract

Gender affirmation surgery (GAS) is a collection of surgical procedures that involve the reconstruction of patients’ genitalia with the aim of achieving the physical appearance and functional abilities of the gender they desire. They are classified into male-to-female (MtF) and female-to-male (FtM). This study is aimed at assessing sexual satisfaction after MtF GAS. A total of 150 patients who underwent MtF GAS at the Urology Group of Southern California and Sinclair Plastic Surgery in Los Angeles, California, were retrospectively surveyed. In total, we received 29 responses, with an average of five years after their surgery. There was a significant correlation between the sensitivity of the neoclitoris and sexual satisfaction but not with vaginal sensitivity or depth. Furthermore, this study revealed an important correlation between gender dysphoria relief after GAS.

## Introduction

Male to female (MtF) transgender women often opt for GAS as part of their transition to address their gender dysphoria. The Diagnostic and Statistical Manual of Mental Disorders, Fifth Edition (DSM-V) of mental disorders defines gender dysphoria as a “marked incongruence between one’s experienced/expressed gender and assigned gender” [1]. Oftentimes, due to this discrepancy, transgender individuals experience immense psychosocial stress, leading them to seek out ways to alleviate the disconnect. Since the healthcare community has started to recognize such feelings, medical professionals have researched sources of treatment for these individuals. Some of these therapeutic options include psychotherapy, hormonal therapy, surgery, or a combination of all three [2]. As the transgender population continues to grow, more people are opting for surgical treatment with the advancement of technology and more permanent options [3]. One procedure common among MtF women is vaginoplasty, in which the penile and scrotal genitalia are modified into a structure that appears and functions similar to that of a cis women’s vagina [4]. The aim of this procedure is to ultimately allow the individual to experience sexual satisfaction as their preferred gender. Previous studies have shown that GAS procedures have positive effects on transgender women [5-6].

Patients who perceived themselves completely as women after GAS have reported higher rates of satisfaction with their current lives [6]. GAS procedures have been proven to help alleviate gender dysphoria and improve mental health. However, little is known about the effects of GAS on MtF women’s sexual satisfaction in the United States. One study conducted by Hess et al. examined the effects of GAS on sexual satisfaction in a German population [7]. The goal of this study was to corroborate the results of Hess et al. and determine the effects of GAS on sexual satisfaction in MtF transgender women in an American population with emphasis on sexual arousal and orgasm quality after surgery.

## Materials and methods

To conduct this study, patients who had undergone a vaginoplasty at the Urology Group of Southern California and Sinclair Plastic Surgery between 2016 and 2019 were contacted via telephone. One hundred and one (101) patients were called and asked if they would like to participate in an online anonymous survey that would be provided to them via email. Thirty-nine (39) showed interest in participation and provided their updated email address to receive the online survey, and 10 participants opted to fill out the survey over the phone. In this case, survey questions were asked over the phone, and participants’ answers were recorded by the surveyor. However, the patient’s name was not recorded on the survey to maintain complete anonymity. The survey covered topics such as satisfaction with vaginal depth, vaginal sensitivity, clitoral sensitivity, and frequency and intensity of orgasms as well as questions on patient demographics. Responses to the survey were collected, and the data were analyzed using Google Forms.

## Results

Out of the 39 emails sent to participants who provided their updated email address over the phone, 19 participants filled out the entire anonymous online survey regarding their sexual experience and satisfaction post-procedure without question omission and 10 survey responses were recorded over the phone, resulting in a 74.4% (n=29) response rate. To keep the survey anonymous, the patients were asked to choose the ranges that their respective ages fell into. The majority of the patients were aged between 31 and 40 years old. Participants in the 21-30 age range (n=6, 20.7%), 31-40 (n=12, 41.4%), 41-50 (n=2, 6.9%), and 51-60 (n=8, 29.6%). Additionally, participants provided their ethnicity; 55.2% of the patient population identified as White and 27.6% identified as Black or African American.

In order to analyze sexual satisfaction, participants were asked about their temporal engagement in sexual activity after GAS. Over half of the participants engaged in sexual activity less than once a month (55.2%, n=16), whereas eight (27.6%) engaged one to three times a week and five (17.3%) more than three times a week. We asked our patients how easy it was to get aroused when they did participate in sexual activity, measured by self-reported responses. Out of the 29 women who responded, 12 (41%) indicated it was always easy to get sexually aroused. Nine (31%) stated it was mostly easy, for seven (21.4%), it was seldom easy, and for one woman, it was never easy. Participants also rated their enjoyment of sexual activity after GAS. With the transition to female genitalia, 65.5% of women were now able to find sexual activity more pleasurable; only six (20.7%) women found it to be less pleasurable and four (13.8%) experienced no difference in pleasurability.

**Figure 1:**
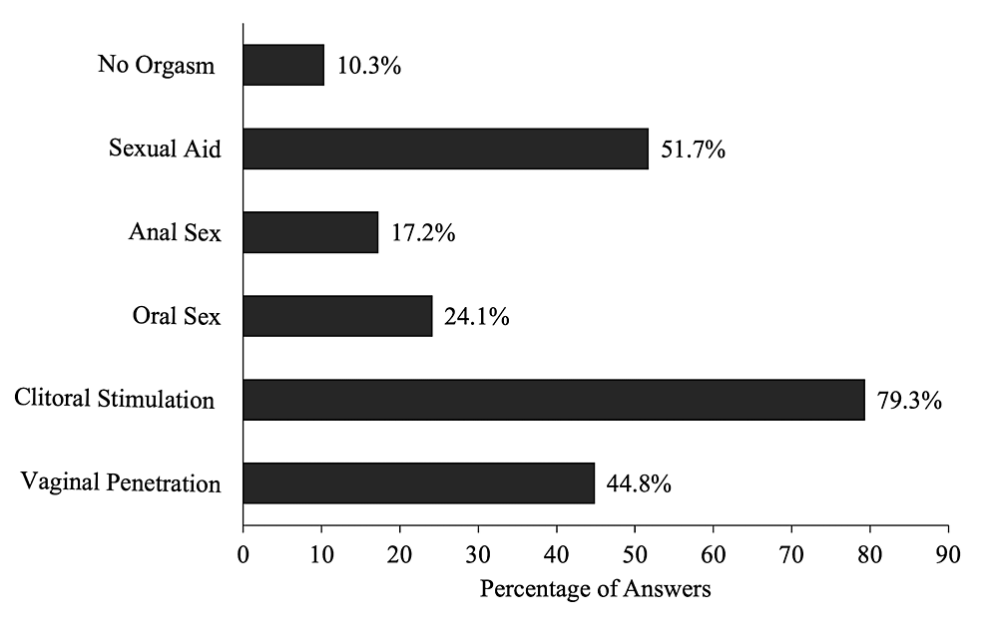
Modality As To How Orgasms Were Achieved (Multiple Answers Possible)

Next, survey participants were asked to compare the frequency and intensity of achieved orgasms before and after GAS. Ten women experienced less frequent orgasms post-op than before surgery, nine out of 29 experienced more frequent orgasms and 10 experienced no change in frequency. The study reported that orgasms in 37.9% (n=11) females were more intense than before surgery, 24.1% (n=7) had less intense orgasms and 37.9% (n=11) saw no difference between before and after. The most popular modality in which women achieved an orgasm was through clitoral stimulation (79.3%) followed by usage of a sexual aid (51.7%) and vaginal penetration (44.8%), as presented in Figure 1. Respondents were able to select multiple modalities through which they achieved an orgasm since surgery. Functional and physical variables such, as vaginal canal depth and sensitivity, clitoral sensitivity, and other postoperative outcomes, can play an imperative role in sexual satisfaction and regret following MtF GAS [7]. The survey asked participants whether or not they measured their vaginal depth and if so, what was the recorded depth in inches. Out of the 29 women who recorded their vaginal depth, the majority (27.6%, n=8) measured 4-5 inches and 20.7% (n=6) measured 5-6 inches. The rest of the women were equally split between not applicable (N/A), under 4 inches, and above 6 inches, 17.2% (n=5) in each respective category.

**Figure 2:**
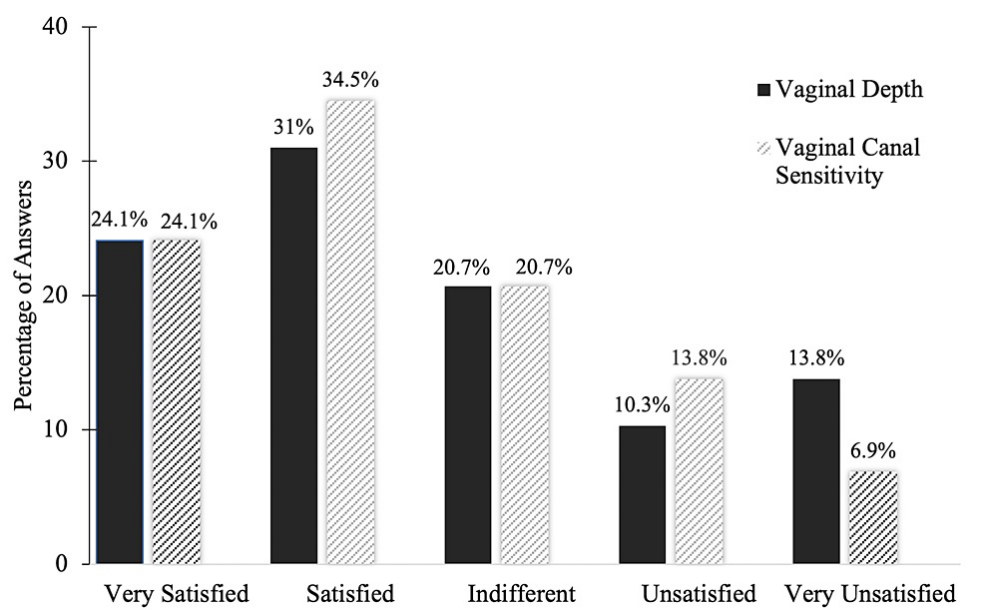
Sexual Satisfaction of Vaginal Depth and Vaginal Canal Sensitivity

**Figure 3:**
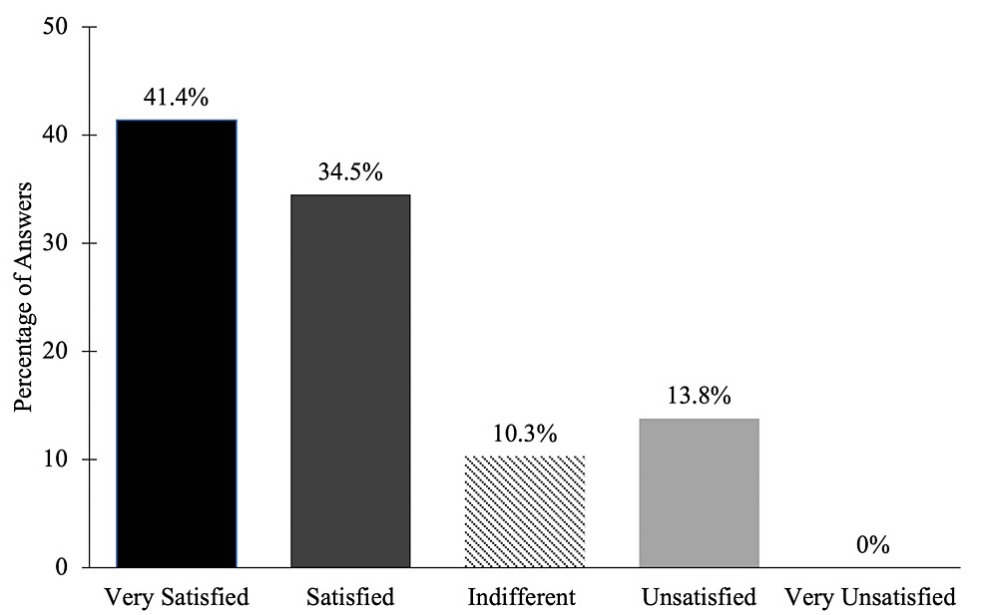
Sexual Satisfaction of Clitoral Sensitivity

**Figure 4:**
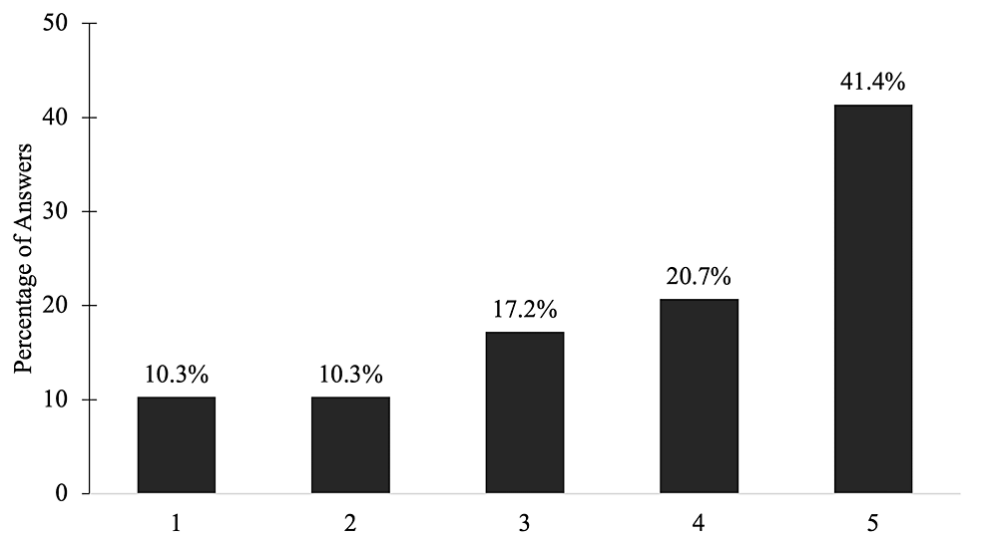
Satisfaction of General Sex Life After Surgery Likert scale ranging from 1 (“Very Dissatisfied”) to 5 (“Very Satisfied”)

After reporting vaginal depth measurements, the women rated their satisfaction with the vaginal depth as well as the neovaginal canal sensitivity, as outlined in Figure 2. Roughly a third of the women were “satisfied” with both parameters, with vaginal canal sensitivity being the most satisfactory. Clitoral sensitivity seemed to play a more pivotal role in sexual satisfaction, with 41.4% (n=12) of women stating they were “very satisfied” (Figure 3). When asked which factor out of all three postoperative variables contributed the most to their enjoyment of sexual activity following GAS, 65.5% (n=19) of women agreed upon clitoral sensitivity over vaginal sensitivity and depth, in which five (17.2%) women chose each of these categories equally. Overall, our cohort was found to have a positive response on their satisfaction with general sex life after surgery. By using the Likert scale, women rated their satisfaction on a scale ranging from 1 (“very dissatisfied) to 5 (“very satisfied”). More than half of the women rated their general sex life with a score of 4 (20.7%, n=6) or 5 (41.4%, n=12), as illustrated in Figure 4. As the goal of many of these participants is to either use psychological interventions or medical modalities as a treatment for their gender dysphoria, we followed up with participants by asking whether or not their gender dysphoria was impacted by undergoing GAS [8]. Twenty-two of the 29 participants (75.9%) indicated their gender dysphoria was decreased as a result of GAS, while 17.2% (n=5) claimed their level of gender dysphoria did not change and 6.9% (n=2) noticed an increase.

## Discussion

Most transgender men and women who met the necessary surgical criteria for GAS experience had improved quality of life, happiness, and sexual function after GAS [9]. Hess et al. found that 91.4% of patients who responded to the questionnaire were very satisfied with their sexual function after GAS [6]. From our findings, an overall sexual satisfaction rate of 79.3% (Likert scale > 3) was observed in our participants (Figure 4). Our article placed a particular emphasis on GAS patients within the United States of America, while the study conducted by Hess et al. focused on the German population. Hence, a high level of patient satisfaction after GAS can therefore be observed and infers that these surgical procedures can function as a key tool to improve sexual satisfaction in the transgender patient.

Our study revealed that 100% of participants identified their gender as female within five years of their GAS. This contrasts with findings from Zavlin et al., who discovered no change in sexual orientation in two distinct age groups, after one year of GAS [10]. This difference may be attributed to the time needed for acclimatization to one’s new anatomy. Therefore, it must be noted that more studies are needed to investigate the psychological and physical factors that influence GAS patients with their gender transition in the United States.

Furthermore, neoclitoral sensitivity was found to be the most important determinant of sexual satisfaction after GAS. From our cohort, 65.5% (n=19) of women agreed that clitoral sensitivity was more determinant of sexual satisfaction versus vaginal sensitivity and depth. This finding parallels that of Hess et al., which reported that the self-estimated pleasure of sexual activity was significantly correlated with the sensitivity of the neoclitoris (p=0.079) [6]. This phenomenon can perhaps be explained by progressive neovaginal desensitization in the post-op period and over time. Our findings can have implications for current gender affirmation procedures since techniques can be implemented to preserve neoclitoral sensitivity. In addition, more studies can be conducted to investigate the major factors that influence neoclitoral sensitivity in the post-op period, to ensure that future patients have a guaranteed positive sexual experience.

Neovaginal depth was also an important element of sexual satisfaction in a GAS patient. The average depth of the neovaginal canal for our cohort was 4-5 inches (27.6%), which fell within the average range for an adult biological female vagina where the mean vaginal length was 9.6 cm (3.78 in) with a wide range, varying from 6.5 cm (2.56 in) to 12.5 cm (4.92 in) [11]. However, the population did report a change in vaginal depth since post-surgical recovery, with 44.8% of patients indicating a decrease in vaginal depth. It seems as though over time, neovaginal depth can be affected by stenosis or ineffective dilatation by the patient postoperatively [6]. In the questionnaire, our participants were allowed to elaborate on any complication they experienced after surgery; a few patients commented on “difficulty dilating due to pain” and “loss of depth.” Postoperatively, 89.7% of patients stated they “mostly” followed postoperative care directions, however, 10.3% stated they “somewhat” followed directions. Consequently, ineffective dilatation or inappropriate use of vaginal dilators could be key factors contributing to neovaginal depth loss being less sexually satisfying.

In addition, it was noted that 65% of women found sexual activity more pleasurable, with 37.9% reporting more intense orgasms after GAS. A systematic review, conducted by Andréasson et al., reported that 67% of respondents were able to achieve orgasm within six months of surgery [12]. However, the ability to attain orgasms after GAS is determined by several factors such as swelling, pain, hematomas, etc. Intuitively, these complications may prevent patients from attempting penetrative or nonpenetrative sexual intercourse, in which case, orgasm may be delayed or nonexistent postoperatively. 

As mentioned previously, gender dysphoria is defined as the marked incongruence between one’s experienced or expressed gender and assigned gender [13]. From our cohort, 75.9 % of participants reported that their gender dysphoria improved after GAS. There are more than eight million adults in the United States who are Lesbian, Gay, or Bisexual (LGB), comprising 3.5% of the adult population, with homosexual and bisexual orientations consisting of 1.7% and 1.8%, respectively [14]. Hence, the prevalence of the growing LGBTQ community places great significance on our study since GAS can be used to address underlying gender dysphoria. Although not investigated in our study, there are several factors that can hinder the resolution of gender dysphoria such as post-op complications or a history of physical or sexual abuse. More research on gender dysphoria outcomes in GAS patients is needed to further enhance their quality of life. We encourage further research to be conducted in the United States analyzing the functionality of the neovagina with post-GAS sexual satisfaction metrics.

## Conclusions

This study was limited by a response rate of 74.4%, which resulted in a sample size of 29. Thus, findings may have been influenced by the responses lost to follow-up. Another limitation of this study is recall bias, as the participants may have had difficulty recollecting experiences several years after their surgery. More long-term follow-up is needed for these MtF GAS patients, as sexual satisfaction may change as time progresses. Another possible limitation is the standardization of questionnaires, as open-ended and close-ended questions among different studies can alter participant responses.

This is the first study that focused on the sexual satisfaction of MtF GAS patients within the United States of America. To the best of our knowledge, no prior research has assessed patient satisfaction to this level of detail. From our findings, the most important element responsible for sexual satisfaction in MtF GAS patients is neoclitoral sensitivity. Our patients also reported a greater relief in gender dysphoria with more enjoyable sexual activity and intensified orgasms after GAS.
