# Correction: Sexual Satisfaction After Gender Affirmation Surgery in Transgender Individuals

**DOI:** 10.7759/cureus.c70

**Published:** 2022-08-11

**Authors:** Renard R Jerome, Maneesha K Randhawa, John Kowalczyk, Alexander Sinclair, Ishita Monga

**Affiliations:** 1 Internal Medicine, St. George's University School of Medicine, St. George, GRD; 2 Psychiatry, St. George's University School of Medicine, St. George, GRD; 3 Surgery, Southern California Hospital, Los Angeles, USA; 4 Biological Sciences, University of California Los Angeles, Los Angeles, USA

This article has been corrected due to the presence of incorrect and misleading definitions and statements related to gender identity and sexual orientation. The corrections did not affect the primary focus of the study (evaluating sexual satisfaction), thus the decision was made to move forward with a correction as opposed to a retraction. The journal deeply regrets that these issues were not identified and corrected prior to publication. A detailed comparison of the uncorrected and corrected versions is available from the journal upon request.

The authors have provided the following statement to accompany the corrected article:

"The main purpose of our study was to evaluate sexual satisfaction in our patient population. Questions about sexual orientation were included for additional information. After input from the public and the journal, we are aware some of the questions were wrongly worded and not conducive to what the overall study is trying to convey. We decided to omit several sections to focus on our main findings which include assessing ability to achieve orgasms, frequency and intensity of orgasms, as well as, self-reported gender dysphoria relief."

